# High throughput mutagenesis and screening for yeast engineering

**DOI:** 10.1186/s13036-022-00315-7

**Published:** 2022-12-27

**Authors:** Kendreze Holland, John Blazeck

**Affiliations:** 1 Department of Biomedical Engineering, Georgia Institute of Technology and Emory University, Atlanta, Georgia USA; 2 Bioengineering Program, Georgia Institute of Technology, Atlanta, Georgia USA; 3 School of Chemical and Biomolecular Engineering, Georgia Institute of Technology, Atlanta, Georgia USA

**Keywords:** High throughput, Directed evolution, Selection schemes, *S. cerevisiae*, Yeast-display, *In vivo* mutagenesis, CRISPR-Cas9, Genome-wide, Cellular engineering, Protein engineering

## Abstract

The eukaryotic yeast *Saccharomyces cerevisiae* is a model host utilized for whole cell biocatalytic conversions, protein evolution, and scientific inquiries into the pathogenesis of human disease. Over the past decade, the scale and pace of such studies has drastically increased alongside the advent of novel tools for both genome-wide studies and targeted genetic mutagenesis. In this review, we will detail past and present (e.g., CRISPR/Cas) genome-scale screening platforms, typically employed in the context of growth-based selections for improved whole cell phenotype or for mechanistic interrogations. We will further highlight recent advances that enable the rapid and often continuous evolution of biomolecules with improved function. Additionally, we will detail the corresponding advances in high throughput selection and screening strategies that are essential for assessing or isolating cellular and protein improvements. Finally, we will describe how future developments can continue to advance yeast high throughput engineering.

## Background and introduction

The ability to engineer, screen, and construct mutant libraries at high throughput is a requisite for maximizing the use of microorganisms for biocatalytic conversions and evolutionary studies. To this end, several techniques have been developed that allow for scalable genetic control, e.g., gene knockouts and transcriptional control strategies, as well as directed evolution of specific genetic sequences. Moreover, advances towards genome-wide studies have allowed for high throughput characterization of yeast genetics, with applications in cell and gene therapies, diagnostics, and biofuels [1–3].

The single-celled eukaryote, *Saccharomyces cerevisiae,* is a platform organism, in part due the development of engineering strategies that allow probing of its entire genome in a high throughput manner. For instance, the yeast genome knockout database has allowed for rapid screening of gene-dependent fitness effects, as well as development of derivative technologies for creation of genetic interaction maps (using pairwise deletion libraries) and chemogenomic platforms that identify chemical compounds specific for yeast target genes (by using haploinsufficiency and homozygous profiling) [4–6]. In addition, more recent strategies such as CRISPR/Cas9 genome editing and CRISPR/dCas9 transcriptional control have significantly reduced the time and labor required to analyze a protein of interest in any genetic background. These tools have been implemented in a variety of applications, including increasing biochemical production (e.g., ethanol [7–12], n-butanol [10], or fatty acids [10–12]) and improving protein secretion (e.g., α-amylase [13]).

In this review, we will highlight conventional and emergent genome-scale and targeted engineering efforts developed for *S. cerevisiae*. Yeast-based high throughput techniques have revealed valuable information pertaining to genotype to phenotype relationships, as well as enabled yeast to become a platform for engineering biomolecules with improved function. Additionally, we’ll discuss the high throughput selection and screening schemes that are often essential for assessing a large, diverse population of engineered strains or selecting improved biomolecules, including techniques that allow continuous evolution of protein sequences. Finally, we will describe how future developments will continue to advance yeast high throughput engineering.

## Genome-wide perturbation strategies for dissecting gene function and improving phenotype

Decades ago, ease of targeted gene deletions in *S. cerevisiae* beget an era of high throughput genome-wide studies to identify gene essentiality [14], protein-small molecule interactions [15], and fitness benefits in response to changing environmental conditions [16]. These studies were soon complemented by overexpression libraries to dissect the impact of constitutive or inducible transcriptional upregulation of each gene in the yeast genome [17, 18]. More recently, the implementation of CRISPR/Cas techniques in yeast have expanded these genome-wide studies to allow screening of gene deletion, overexpression, or inhibition via a single platform [19]. Cataloguing previously generated information, the *Saccharomyces* Genome Database (SGD) provides a comprehensive list of yeast genes and associated phenotypes, functions, and interactions [20], and other databases detailing the yeast proteome, protein phosphorylation sites, and molecular interactions also provide accessible information [21–25].

### Yeast gene deletion collections for genome-wide fitness characterizations

*S. cerevisiae* has an extremely efficient ability to perform homologous recombination (HR) mediated insertion of DNA into its genome, allowing for facile construction of strains with specific gene deletions [26]. The yeast deletion collection comprises a library of *S. cerevisiae* strains in which the large majority of open reading frames in the yeast genome have been knocked out (one deletion per strain) [27, 28]. This resource was driven by the complete sequencing of the yeast genome, and it expanded upon prior deletion projects in which less controllable mutation strategies were utilized, such as incorporation of mini-transposons or Ty1 [29–31]. Using fitness based screens of a deletion collection, the essentiality of genes has been determined in rich and minimal media, as well as the requirement of genes for optimal growth in a variety of other common growth conditions (e.g., high salt, galactose carbon source) [27, 28]. The yeast deletion collection has also been shown to be a valuable tool for functional genomics characterizations, for instance for genes involved in nucleotide excision repair and DNA repair after UV damage, cell cycle checkpoints, and homologous recombination [32].

**Fig. 1:**
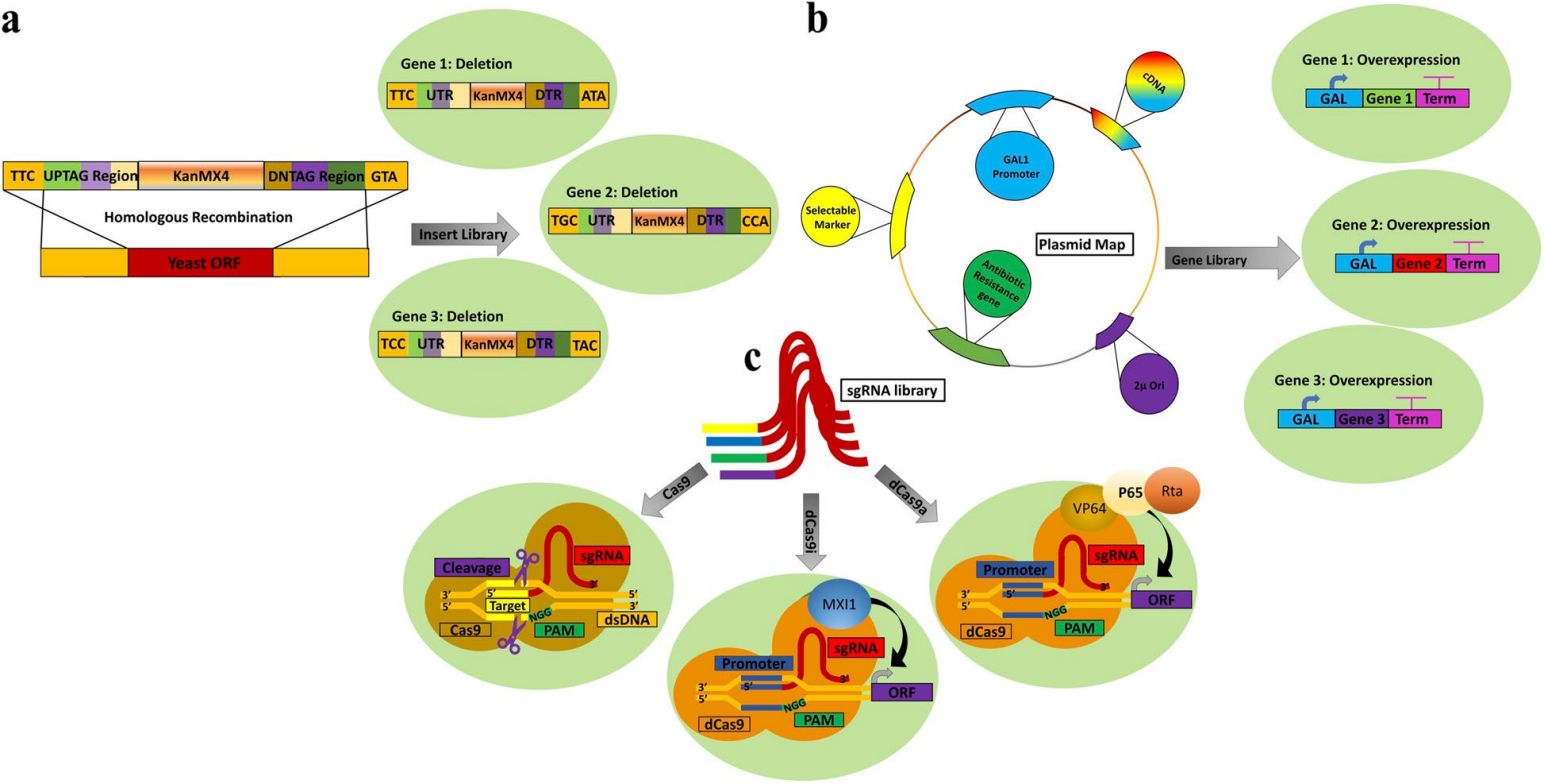
Genome-wide screening strategies and collections. **a** Gene deletion libraries can be constructed using homologous recombination mediation insertion of tagged antibiotic resistance genes (e.g., KanMX4) in place of yeast genes. **b** Overexpression genomic libraries can be created by placing genomic or cDNA libraries under control of strong (e.g., the inducible GAL1) promoters (GAL1) on a large collection of plasmids and introducing them into yeast cells. **c** Insertion of a library of sgRNAs into yeast cells that have been engineered to expressed Cas9, dCas9-MXI1, or dCas9-VPR can enable gene deletion, expression inhibition, or transcriptional activation, respectively. The impact of these genetic perturbations can be screened with an appropriate high throughput set up

To enable genome-wide functional characterization of genetic interactions, i.e., the impact of one gene on another and vice versa, a remarkable collection of 23 million yeast strains with two gene deletions per strain has been created, which allowed characterization of ~550,000 negative (reduced fitness compared to single deletions) and ~350,000 positive (increased fitness compared to single deletions) genetic interactions [33]. This effort was further expanded through construction of ~200000 yeast strain with triple gene deletions and partial characterization of ‘trigenic’ interactions [34]. Overall, yeast deletion collections have provided invaluable information about the yeast genome by easing high throughput evaluations. For general yeast genomic engineering strategies, deletion collections can be utilized as a tool to ease the knockout of a given gene in a new yeast strain, through HR-mediated introduction of an amplified deletion cassette (Fig. 1a).

### Overexpression gene libraries for complementary genome-wide characterizations

Gene overexpression libraries provide valuable tool for cellular engineering and screening altered yeast phenotypes, including resistance to inhibitory environmental conditions, that is complementary to deletion collections (Fig. 1b). In an early example, 24 overexpression (OE)-sensitive clones, engineered by inserting a complementary DNA (cDNA) library (1.5*10^6^ clones) in a multicopy vector under the control of the inducible GAL1 promoter, resulted in rapid growth arrest of yeast cells in the G1 or S stage, allowing identification of cell proliferation regulators [35]. These OE-sensitive hits included MCM1, a transcription factor for mating type-specific genes, twelve other genes with known function, and eleven uncharacterized Src Homology Domain Containing E (SHE) genes, which are now known to inhibit dynein function (SHE1) or interact with She3p (SHE2), impacting cellular growth and development [35–37]. A separate overexpression library screen showed the reverse phenotype for ferritin, a protein important for iron regulation and production of free oxygen radicals, which increases yeast replicative lifespan. The same work demonstrated that overexpression of genes involved in ubiquination (UBC3, UBC4, UBC5, and UBC7) increased yeast survival when exposed to methylmercury [38]. Similarly, genes that improve yeast resistance to cadmium (e.g., CAD1, CUP1) were identified using an overexpression library [39]. An overexpression screen using a collection of 7,777 plasmids covering 97.2% yeast genome identified genes (YLR247c, RAD26, PSH1) and gene fragments (N terminus of POB3) that induced Bur^-^ (unable to grow on media containing sucrose) and Spt^-^ (unable to grow on media lacking lysine) phenotypes [40].

Interestingly, a genome wide overexpression screen with yeast and a genomic DNA library (average insert size, 5kb) allowed identification of small-molecule effector and inhibitors of cellular processes (e.g., CGP60474 (an inhibitor of CDK1)), demonstrating that yeast functional genomics via OE-libraries can be used to rapidly identify cellular targets of small molecules [41]. Moreover, a genome-wide library of strains carrying ‘SWAp-Tags,’ in which each protein has a NOP1promoter-GFP module at its N’ terminus, has enabled easier exploration of protein abundance, localization, topology, and interactions with other proteins [42]. And recently, the YETI (Yeast Estradiol strains with Titratable induction) collection was developed, which consists of a suite of >5,600 yeast strains that allow transcriptional upregulation of genes of interest in response to β-estradiol [17].

OE-libraries do not have to be limited to characterizing the impact of native *S. cerevisiae* gene overexpression*.* An inverse metabolic engineering methodology screened for the ability of *Pichia stipites* (*Scheffersomyces stipites*) genomic DNA fragments to improve *S. cerevisiae* growth in xylose-based media, identifying the XYL1, XYL2, XYL3, and PsTAL1 genes [43]. In general, OE-libraries enable large-scale systematic analysis of mutant phenotypes that can be expanded to dissect gene function in a genome-wide manner. Of note, while gene deletion collections may not allow characterization of essential genes, OE-libraries might allow investigation into their function using an inverse approach. As we discuss below, promising platforms have recently been developed for genome-wide overexpression and knockdown/deletion using CRISPR-Cas-aided engineering, allowing direct coupling of these two strategies [44–46].

## CRISPR/(d)Cas-mediated genome-wide screens for rapid and multiplex assays

The recent developments of CRISPR/(d)Cas technologies have enabled rapid generation of genetic deletions and facilitated high throughput transcriptional perturbations screens across the domains of life, including in *S. cerevisiae* [47–49]. The CRISPR (cluster regularly interspaced short palindromic repeats)/Cas9 system employs an RNA-targeted Cas9 protein with exquisite targeting capabilities that are retained even when its native endonuclease activity is abrogated (i.e., deactivated Cas9 or dCas9) (Fig. 1c). Other targeted endonucleases (e.g., Cas12) can also function effectively in yeast [50].

### CRISPR/Cas-mediated gene knockout studies

To demonstrated functionality of the bacterial CRISPR/Cas9 system in yeast, DiCarlo *et al*. co-expressed a single-guide RNA (sgRNA) and Cas9 to mutate CAN1, an arginine permease, where its mutation makes cells resistant to the toxic arginine analogue, canavanine [51]. They further showed that Cas9-directed DNA double stranded breaks greatly improved HR-mediated insertion of donor DNA [51]. Such single-target Cas9-mediated genetic knockouts are now used routinely in yeast, for instance to improve bioethanol production processes [8, 52]. CRISPR/Cas9 has also been used to generate genome-wide knockout libraries in *S. cerevisiae.* For instance, the homology directed-repair-assisted genome-scale engineering (CHAnGE) method, developed for targeted mutation, was validated by generating a large deletion collection that was screened for furfural tolerance [53]. CRISPR-Cas techniques can also be expanded to simultaneously targeting multiple genes in one experiment. For instance, a sgRNA-tRNA array allowed editing of up to 8 genes at a time with 87% efficiency in yeast [11].

### (d)Cas9-mediated transcriptional control and multifunctional genome-wide assays

Transcriptional inhibition can be mediated by deactivated Cas9 in yeast (CRISPRi), with greater repression when targeting the 200 bp region immediately upstream of the transition start site, as well as when targeting nucleosome-depleted and open chromatin regions [54]. Inhibition potency can also be improved through fusion with repressor domains, such as MXI1 [55]. In a genome-wide study, Momen-Roknabadi and colleagues developed an anhydrotetracycline inducible CRISPRi screen, surveying *S. cerevisiae* genes with >51,000 sgRNAs to dissect haploinsuffiency and genes involved in adenine and arginine biosynthesis [56].

Similar to CRISRi, CRISPRa utilizes (d)Cas9 targeting of promoter regions, but fused to VPR (VP64-p65-Rta) or other transcriptional activators to increase gene expression [57]. Dong *et al.* developed a clever three-way gene control schema, utilizing a dCas9 protein for CRISPRi and demonstrating that with only one protein (Cas9-VPR), it was possible to either activate or mutate gene targets, depending on gRNA length [45]. Such efforts for trifunctional genetic control in yeast cells were furthered by utilizing three orthogonal (d)Cas9 proteins or protein fusions for activation, deletion, or inhibition (dubbed CRISPR-AID), which could be used at the genome-wide level by using them in conjunction with oligonucleotide arrays [19, 46]. Trifunctional control using these systems has been used to augment α-santalene biosynthesis, improve Beta-Carotene production, enhance yeast surface display, and to identify uncharacterized genetic determinants of complex phenotypes (i.e., furfural tolerance) [19, 45, 46]. CRISPR-Cas-aided genome-wide overexpression and knockdown was also used to increase isobutanol titers and enhance cell growth in glycerol [44].

## Targeted diversification strategies for protein or pathway evolution

Evolution of proteins or biological pathways can be achieved by multiple rounds of diversification *in vitro* (benchtop) or continuous evolution (*in vivo*) of specific gene targets with selection screens built for desired phenotypes. In the next sections, we will discuss the methods involved in generating targeted biological diversity for testing using yeast or directly within yeast at high throughput.

### *In vitro* techniques for generating biological diversity for yeast optimization

Large mutant libraries of specific DNA sequences with varying properties can be generated by traditional methods such as random substitution mutagenesis (e.g., error prone PCR (EP-PCR)) or DNA shuffling, and then screened in yeast [58–60]. For instance, EP-PCR of the SPT8 gene (suppressor of ty insertion 8) and screening allowed isolation of mutants that afforded 8.9% higher ethanol tolerance and 10.8% higher ethanol production for yeast strains than the wild-type gene, and DNA shuffling of endoglucanase I genes from three *Trichoderma* sp. (*T. reesei, T. pseudokoningii* and *T. longibrachiatum*) and screening allowed isolation of variants with improved activity [61, 62]. These traditional mutagenesis techniques are also often coupled to yeast surface display to select for high affinity binders and other enhanced protein properties [63, 64].

### Targeted *in vivo* CRISPR-mediated diversification

Directed evolution experiments that involve CRISPR/Cas-based mutagenesis can diversify targeted DNA sequences, often taking advantage of native yeast molecular machinery (primarily HR-insertion of mutant DNA) to engineer metabolic pathways and improved stress tolerance. For example, the Multiplex CRISPR (CRISPRm) system used EP-PCR to create a library of mutant cellodextrin transporters, which was transformed with overlapping promoter and terminator DNA for assembly and genomic integration. A mutant cdt-1 afforded 2.6-fold improved cellobiose utilization compared to the wildtype transporter [65].

In yeast, biological diversity can also be generated directly *in vivo*. For instance, CHAnGE, in which gene edits are tracked by guide RNAs linked to homologous repair cassettes, has allowed for genome-wide engineering of *S. cerevisiae* at high throughput [53, 66]. A CHAnGE plasmid library (between 1.2 x 10^7^ and 4 x 10^7^ members) transformed into yeast allowed screening of disruption mutants for improved resistance to furfural. Selected mutants with improved phenotypes displayed an enrichment in SIZ1, SAP30, UBC4, and LCB3 targeting guides. In an attempt to reduce toxicity issues during alcohol production, Liang *et al*. targeted 25 regulatory genes for mutation using the multiplex navigation of global regulatory networks (MINR) method, isolating a variant of the WAR1 transcription factor that enabled higher tolerance to both isopropanol (50-60 g/L) and isobutanol (14-16 g/L) [53, 66–68]. In addition, Cas9-mediated protein evolution (CasPER), which can be used for selection marker-free integration of large (>120 bp) DNA fragment into the yeast genome, was used to improve mevalonate pathway flux and downstream carotenoid production by 11-fold [69].

### Targeted *in vivo* continuous evolution

**Fig. 2:**
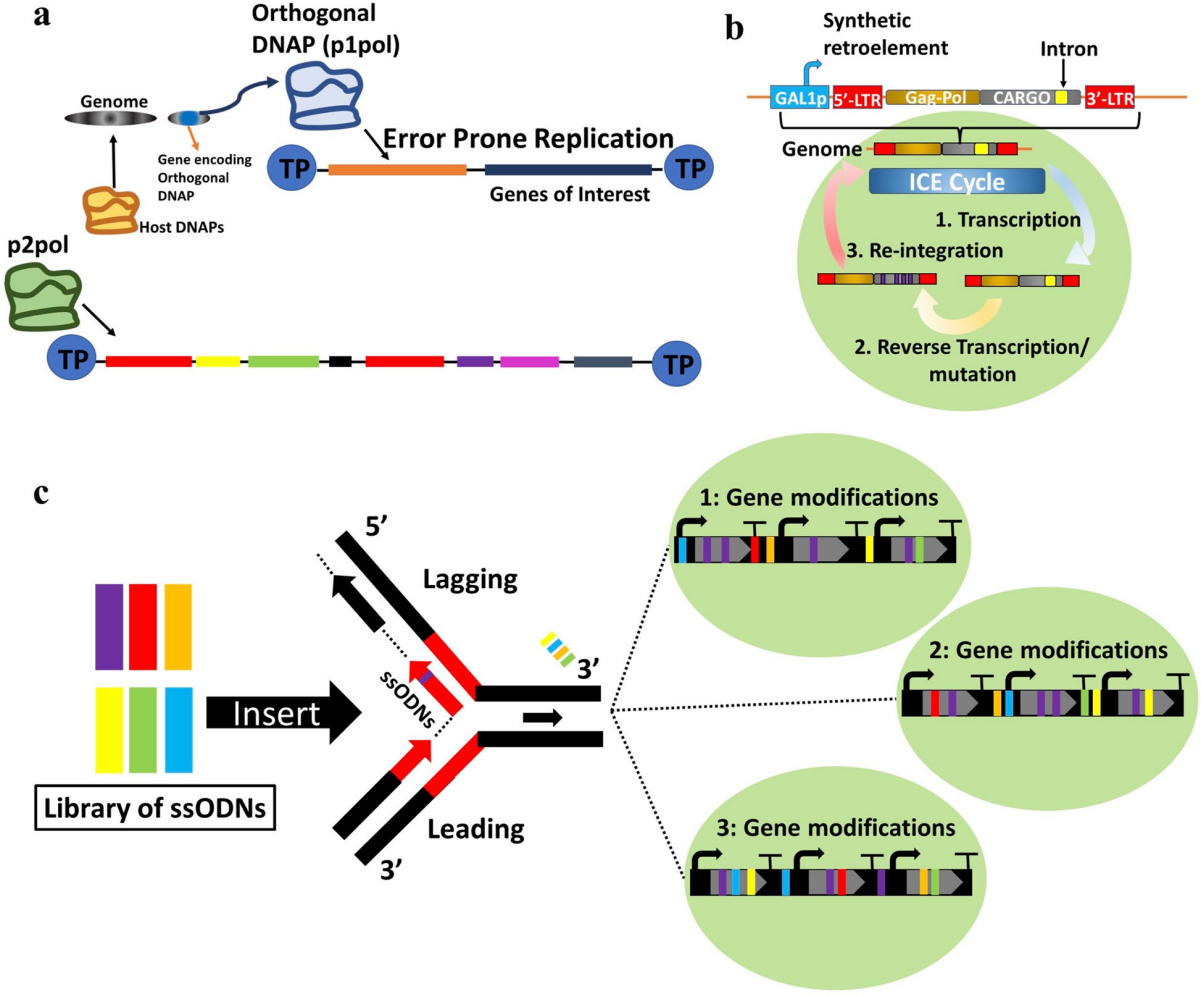
*In vivo* continuous evolution strategies. **a** OrthoRep for error-prone and orthogonal DNA replication; The error-prone DNA polymerase (DNAP) (p1pol) does not interact with the host genome, instead mutating only genes of interest during their replication. DNA polymerase (p2pol) also replicates a needed accessory plasmid. **b** *In vivo* continuous evolution (ICE) method for reverse-transcription based mutagenesis in three steps; (1) Genetic cargo and other elements are flanked by long terminal repeats (LTR) of a Ty1 retrotransposon and inserted into the genome. (2) After its transcription, the cargo is reverse transcribed by an error-prone reverse transcriptase into cDNA, introducing mutations. (3) The mutated cargo cDNA is integrated into the genome. **c** Eukaryotic multiplex automated genome engineering in yeast (eMAGE); libraries of ssODNS, which bind and introduce mutations into the lagging strand of DNA in the replication fork, are inserted into yeast cells, such that genes, promoters, etc. can be edited in yeast cells over multiple cycles, leading to a wide range of genetic variation. For these *in vivo* continuous evolution strategies, variants with mutated genotypes are screened for improved phenotypes, and if necessary, mutated further over more evolutionary rounds

In yeast, *in vivo* evolution strategies have been designed that can enable continuous targeted diversification of DNA sequences for evolution of a desired protein or cellular phenotype, while circumventing labor and time-intensive DNA extraction, mutation, and transformation steps [70]. For example, utilizing heterologous DNA polymerase (TP-DNAP)-plasmid pairs, i.e., the linear pGKL1/2 plasmids from *Kluveromyces lactis*, allowed creation of OrthoRep, an extrachromosomal orthogonal error-prone replication system, in which genes of interest can be encoded on a *K. lactis* plasmid and mutated continuously during its replication (Fig. 2a). Increasing the mutation rate afforded by a DNAP (a Y427A variant), current DNAPs are known to have per-base substitution mutations ∼100,000-fold greater than the genome [71, 72]. Orthorep was recently leveraged to develop an ‘autonomous hypermutation yeast surface display’ (AHEAD) system to continuously evolve synthetic recombinant nanobodies, for instance, to bind the receptor-binding domain of the SARS-CoV-2 spike protein with high affinity, and to engineer the *Thermotoga maritima* tryptophan synthase β-subunit for enhanced activity or promiscuity [73, 74].

Moreover, Crook and colleagues developed the ‘*in vivo* continuous evolution’ (ICE) platform for targeted and continuous evolution of genes and pathways in yeast [75]. The ICE system employs the retrotransposon Ty1 and an error-prone reverse transcriptase allowing reintegration of mutated complementary DNA in the yeast genome for continuous evolution (Fig. 2b). As proof of concept, ICE was applied to single proteins and multi-protein pathways. For instant, evolution of the Spt15 global transcriptional regular (and its promoter region) allowed for selection of a cassette that enabled improved 1-butanol tolerance, and evolution of a multi-gene xylose utilization pathway afforded a 21% increase in exponential growth [75].

Finally, the eukaryotic multiplex automated genome engineering (eMAGE) system allows for incorporation synthetic single-stranded oligodeoxynucleotides (ssODNs) for targeted and continuous genomic editing (Fig. 2c) [76]. eMAGE was shown to allow for precise gene editing and to afford complex (10^5^) diversity across multiple loci. For instance, using a pool of ssODNs to target various genetic elements in a heterologous β-carotene pathway produced noticeable phenotypic alterations, including enhanced β-carotene accumulation.

## High throughput selection schema for enhanced biological functions

Methods for generating biological diversity must be coupled to similarly high throughput screening strategizes. Thus, several high throughput selection or screening methods that allow for the testing of large quantities (i.e., millions) of mutant variants have been developed to allow selection for enhanced phenotypes [77]. Growth-based selections, biosensors, drop-based microfluidics, and surface display are commonly utilized high throughput techniques for selecting improved yeast cell phenotypes and isolation of optimized biomolecules. Subjecting a large population to high throughput screening and selection can also generate significant new information in a single experiment.

### Growth based selections

**Fig. 3:**
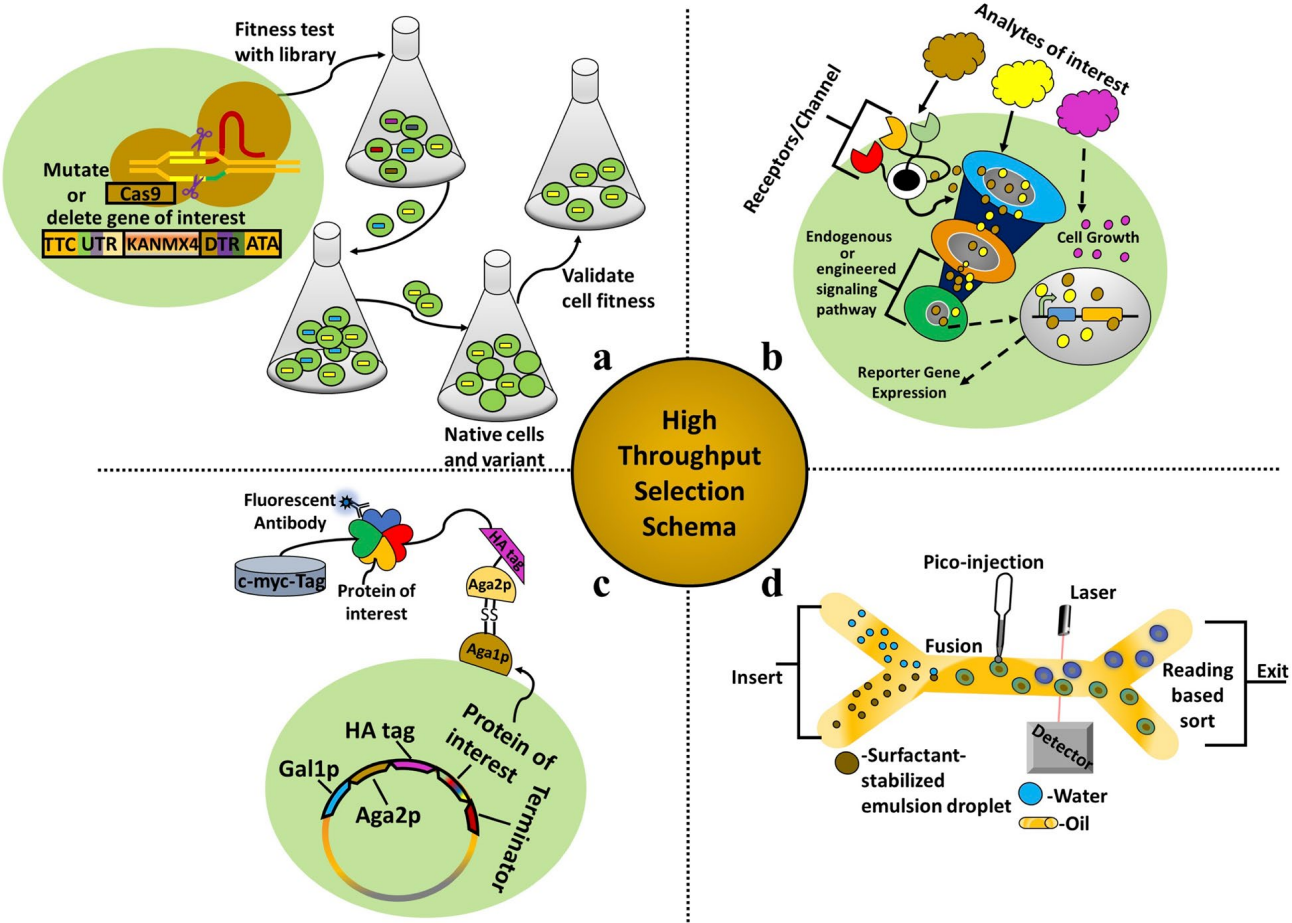
Schematic representation of high throughput selection schemes in yeast. **a** Growth based; Strains with improved fitness (in terms of growth rate) naturally outcompete slower growing strains in batch cultures with successive dilutions. **b** Biosensor; Analyte concentration can stimulate a dose-responsive change in cell growth or reporter gene expression. **c** Yeast surface display; Proteins of interest are secreted but bound to the yeast cell surface due to their fusion to the Aga2 protein. While anchored to the cell surface, the protein of interest is screened using fluorescent activated cell sorting (shown) or another method (e.g., magnetic bead separations). **d** Drop-based microfluidics; Diverse populations of single yeast cells, combined with a stabilizer and aqueous drops disseminated in oil, are screened at high throughput based on detector readings

Growth-based selection does not require expensive equipment or meticulous quantification methods to find links between a genotype and phenotype. Instead, more rapid yeast growth is indicative of an improved protein, pathway, or combination thereof, such that by simply allowing an adequate number of cell divisions, a sub-selection of yeast cells harboring improved variants can be isolated from a larger library (Fig. 3a). In this manner, the activity of enzymes or small molecular transporters within specific metabolic pathways can often be coupled to growth rates. For instance, Lee *et al*. screened a randomly mutated xylose isomerase library for increased growth that would be associated with improved xylose catabolism, isolating an enzyme variant with a 77% increase in catalytic activity [78].

Alternatively, yeast strains can be grown independently to detect sensitivity to environmental conditions, often using a robotic handling set up. For example, growth-based selections of yeast deletion collections in supplemented media have allowed identification of hundreds of gene disruptions that result in sensitivity to ethanol, 1-propanol and 1-pentanol [79, 80]. Similarly, performing a growth-based selection competition in media containing 50 mM furfural on a barcoded *S. cerevisiae* library containing 4848 complete gene disruptions allowed identification of 229 deletion strains with heightened furfural sensitivity, many with disrupted pentose pathway genes (e.g., ZWF1, GND1, RPE1, TKL1) [81]. Growth assays may also be used for phenotypic profiling, where the rate at which the engineered yeast cells grow is quantified to infer the importance of a deleted or mutated gene.

### Biosensors

Biosensors have enabled high throughput analysis of engineered microbial factories, by being able to sense and report the intracellular level of a wide range of otherwise unmeasurable analytes (Fig. 3b). By performing these functions, biosensors have proven invaluable for increasing the production of bio-based value-added chemicals, while helping to prevent metabolic imbalances and accumulation of toxic metabolites [82]. A general fluorescent biosensor database also exists and may help guide future biosensor construction efforts in yeast [83]. We will discuss three classes of yeast biosensors: transcription factor (TF) based, RNA-based, and enzyme-coupled.

When activated by a molecule, certain TFs (either endogenous or heterologous) can be induced to bind specific DNA sequences to activate transcription [84–86]. By coupling these TFs with promoters that (1) contain corresponding TF-binding sites and (2) direct transcription of a reporter protein, it is possible to create a biosensor responsive to a diverse range of analytes [87–91]. For instance, a malonyl-CoA biosensor that utilized the *B. subtilis* TF FapR and its corresponding operator allowed screening of a genome-wide overexpression library and identification of two genes, TPI1 and PMP1, that increased intracellular malonyl-CoA in yeast [84]. A similar biosensor was developed using the Haa1 TF protein from *Bacillus megaterium* to sense acetic acid [92].

RNA-based biosensors employ noncoding mRNA sequences to sense molecules like metabolites or antibiotics [93, 94]. Riboswitch sensors are RNA aptamers, with hairpin-like structures that when bound by a ligand, induce a conformational change in the mRNA tertiary structure, thus acting as a means of controlling translation [94]. Several efforts have demonstrated that these RNA-switches can function in *S. cerevisiae*, though to date, they have not been used for high throughput screening applications in yeast.

Finally, enzyme-coupled biosensors employ enzyme-catalyzed reactions of metabolites of interest into measurable chromogenic or fluorogenic products. For example, the activity of tyrosine hydrolases was screened in yeast using a DOPA dioxygenase-based enzyme-coupled biosensor that converts L-DOPA (the product of tyrosine hydrolases) into the fluorogenic betaxanthin compound [95, 96]. Biosensors have long been applied to select for strains with enhanced production of a given metabolite or flux through a specific metabolic pathway, as well as to perform more genome-wide selections for improved phenotypes. For instance, Wang and colleagues constructed a biosensor-aided screen for *cis, cis*-muconic acid and protocatechuic acid import by engineering promoters to harbor binding sites for their transcriptional regulators and drive expression of a GFP reporter [97]. They further used CRISPR-mediated gene disruptions to identify transporters that influenced the production of these acids.

### Yeast surface display

Yeast surface display, in which a protein or library of protein variants are secreted and affixed to the yeast cell surface via fusion to the Aga2 protein (Fig. 3c), was first demonstrated by Boder and Wittrup in 1997, and used to screen for enhanced affinity of an antibody fragment (scFv) [63]. Currently, yeast surface display is a foundational technology for bioengineering, widely applied for scFv and other protein engineering efforts, as reviewed recently by Teymennet-Ramirez *et al.* [98]. Surface display allows for screening of incredibly large libraires in conjunction with fluorescent activated cell sorting for binding to fluorophore-tagged targets or when using antigen-coated magnetic bead separations. For instance, Feldhaus *et al.* performed screening for different protein, peptide, and hapten antigen targets of a large (size = 10^9^) library of nonimmune human antibody scFv fragments [99]. Using the same principle, native yeast proteins that aid secretion of single-chain T-cell receptors and scFvs (1.5-fold to 7.9-fold improved secretion, respectively) were revealed by screening a genome-wide overexpression library [100]. And as mentioned above, the AHEAD *in vivo* continuous evolution system employs yeast display to select for improved antigen binding from a nanobody library [74].

Surface display can also aid in selection for protein function other than binding. The YESS (yeast endoplasmic reticulum sequestration screening) system enables engineering of protease specificity [101]. In YESS, a protease mutant library can be co-expressed with a substrate fusion protein that contains a selection substrate sequence, a counterselection substrate sequence, and multiple intervening epitope tag sequences. Novel protease specificities can be selected by screening for the presence or absence of these tag sequences using yeast display. Using YESS, the Tobacco Etch Virus protease (TEV-P) was engineered to prefer a glutamic acid or a histidine at P1 of its canonical ENLYFQ↓S substrate sequence rather than glutamine (Q), resulting in 5,000-fold and 1,100-fold changes in selectivity, respectively [101]. The similar YESS 2.0 system improved the catalytic efficiency of the TEV-P variant by 2.25-fold over the wild-type by modulating both the ratio of TEV-P to substrate and their contact time in the endoplasmic reticulum [102].

### Drop-based microfluidics

Drop-based screening platforms use microfluidic devices that encompass individual yeast cells in aqueous drops disseminated in oil, in effect creating single-cell picoliter-volume reaction vessels, to enable rapid screening of large populations or libraries [103, 104] (Fig. 3d). In an early study, yeast cells that displayed a mutant library of horseradish peroxidase enzymes were screened via drop-base microfluidics by coupling the enzyme to a fluorogenic substate. Impressively, the reaction rates of 10^8^ cells in <150µl volume were screened in <10 hours, and variants with intense signals were isolated and further screened for improved activity [103]. In addition, Abatemarco *et al*. created an RNA-aptamers-in-droplets system, which used ultrahigh-throughput droplet microfluidics and analyte-responsive RNA aptamers grafted to the Spinach aptamer backbone to engineer strains of *S. cerevisiae* with increased production of tyrosine or secretion of recombinant streptavidine, screening millions of droplets in hours [105, 106]. While potentially difficult to set up, drop-based microfluidics can afford a low cost, high throughput approach for screening large libraries.

## Conclusion and future perspectives

Native or nonnative genetic sequences can be optimized using yeast-based methods for enhanced capabilities, thereby improved whole cellular phenotypes or specific protein functions. Importantly, the rate and scale by which these sequences/phenotypes can be perturbed, studied, and isolated has been massively increased by both genome-wide and targeted genetic mutation strategies. The model organism *S. cerevisiae* has enabled such high throughput modifications and downstream screening strategies because of its simple and inexpensive cultivation, well-developed genetic tool kits, and impressive ability to perform HR-mediated genomic editing.

Of course, these diversification efforts and the accompanying benefits towards yeast pathway and general biomolecules can still be improved. For instance, the vast majority of genome-wide libraries target only one gene per cell. In particular, genome-wide, multi-gene CRISPR-aided gene targeting experiments, which would be analogous to the double gene knockout collection, have not been performed to date in yeast [11]. Dual gene transcriptional repression via CRISPRi has been performed at scale in human cells, and might be applied to yeast for either multi-gene inhibition or overexpression libraries to glean more insights into the yeast genetic interaction network [107]. CRISPR-based screens might also be improved by further incorporating Cas orthologues with improved properties and that recognize different PAM sites or recognize orthogonal sgRNA scaffolds into yeast studies [49, 108–110]. For instance, CRISPR-Cas12a(Cpf1)-assisted tag library engineering (CASTLING) has been used to create pooled libraries with high accuracy [111, 112]. Finally, integration of *in silico* genome engineering and protein design tools might guide future library designs, increasing the likelihood of attaining a desired phenotype [113, 114].

High throughput screens might also be improved in the future. For instance, the use of biosensors and riboswitches can be limited by the requirement to create and validate a new sensing module for every new analyte of interest. More modular and easily programmable biosensors would greatly assist high throughput screen development. To this end, multi-parameter designs employing the prokaryotic LysR-type transcriptional regulators were recently used to craft a more general biosensor design framework, with some success [115]. Intriguingly, the OrthoRep system has also been utilized for the evolution of yeast-metabolite biosensors, demonstrating how *in vivo* continuous diversification can be employed to overcome potential roadblocks in high throughput screens [116] . In addition, while they are low cost, the accessibility of droplet-based microfluidics limits their adoption. To this end, a more user-friendly procedure that involves ready-to-use or to-be-assembled set-ups can increase the application of this technique for screening large libraries. At the same time, other methods, either *in vitro* or *in vivo*, that allow high throughput library generation, screening, and directed evolution of phenotypes will continued to be gradually enhanced, likely with continued focus on constructing systems that allow coverage of the entire yeast genome in one experiment.

## Data Availability

Data sharing is not applicable to this article as no datasets were generated or analyzed during the current study.

## Abbreviations

AHEAD: Autonomous hypermutation yeast surface display
CasPER: Cas9-mediated protein evolution
CRISPR: Clustered Regularly Interspaced Short Palindromic Repeats
CRISPRa: CRISPR activation
CRISPR-AID: CRISPR activation, deletion, or inhibition
CASTLING: CRISPR-Cas12a(Cpf1)-assisted tag library engineering
CRISPRi: CRISPR inhibition
cDNA: Complementary DNA
EP-PCR: Error-prone PCR
eMAGE: Eukaryotic multiplex automated genome engineering
CHAnGE: Homology directed-repair-assisted genome-scale engineering
HR: Homologous recombination
ICE: *In vivo* continuous evolution
CRISPRm: Multiplex CRISPR
MINR: Multiplex navigation of global regulatory networks
OE: Overexpression
*S. cerevisiae*: *Saccharomyces cerevisiae*
scFv: Single-chain variable fragment
sgRNA: Single-guide RNA
SHE: Src Homology Domain Containing E
ssODNs: Synthetic single-stranded oligodeoxynucleotides
TEV-P: Tobacco Etch Virus protease
TF: Transcription factor
VPR: VP64-p65-Rta
YESS: Yeast endoplasmic reticulum sequestration screening
YETI: Yeast Estradiol strains with Titratable induction
